# Microalgae as a prospective antibiotic replacement therapy for induced endometritis in rats through regulating inflammatory cytokines

**DOI:** 10.5455/javar.2026.m1008

**Published:** 2026-03-05

**Authors:** Emtenan M. Hanafi, Moetazza M. Alshafei, Heba H. Hozyen, Manal M. Ramadan, Yasser H. A. Saber, Reda M. S. Korany, Reda M. S. Korany, Enas N. Danial, Seham S. Kasem, Naglaa A. Ali, Safaa H. Mohamed, Ghada H. Elsayed

**Affiliations:** 1 Department of Animal Reproduction and AI, NRC, Dokki, Giza, Egypt; 2 Department of Nutrition and Food Sciences, NRC, Dokki, Giza, Egypt; 3 Department of Veterinary Physiology and Biochemistry, Faculty of Veterinary Medicine, Ain Shams University, Cairo, Egypt; 4 Department of Chemistry of Flavor of Aromatic Plants, NRC, Dokki, Giza, Egypt; 5 Department of Pathology, Faculty of Veterinary Medicine, Cairo Univ, Giza, Egypt; 6 Department of Chemistry of Natural and Microbial Products, NRC, Dokki, Giza, Egypt; 7 Department of Hormones, Medical and Clinical Studies Institute, NRC, Dokki, Giza, Egypt; 8 Stem Cell Lab, Centre of Excellence for Advanced Sciences, NRC, Dokki, Giza, Egypt

**Keywords:** *Spirulina platensis*, *Chlorella vulgaris*, Endometritis, inflammatory cytokine genes, immunohistochemistry

## Abstract

**Objectives:**

The purpose of this study was to monitor the antimicrobial role of *Spirulina platensis* (SP) and *Chlorella vulgaris* (CV) against induced endometritis using *E. coli* O157 in Albino rats.

**Materials and Methods:**

After preparation of encapsulated flavoured microalgae, they were mixed into the animal diet at 1%. The antioxidant and antimicrobial activity of the algae was studied. 45 adult female rats were classified into 3 groups (G1, G2, and G3) where each group had 15 rats in number. The first group ate a control basal diet (CD), the second group received CD mixed with SP, and G3 received CD mixed with CV. After 20 days of supplementation, animals were infected with an intravaginal injection of a pathogenic strain of *E. coli* O157 to induce endometritis. Vaginal swabs were collected for colony count estimation and to monitor recovery rate. Heparinized blood samples were collected for analysis of plasma chemistry. Samples of the uterus and spleen were taken for histopathology, immunohistochemistry, and gene expression analysis.

**Results:**

SP and CV exhibit strong antioxidant, antimicrobial, and anti-inflammatory effects, and a high rate of uterine clearance, as indicated by downregulation of target cytokine genes used as indicators of the inflammatory process. Significant elevation in plasma cytokines (*TNF-α, INFγ, IL1β*), especially those administered SP and CV. Group treated with Microalgae showed a high recovery rate, including increased mature ovarian follicles, few follicular cysts, and moderate infiltration of endometrial inflammatory cells.

**Conclusions:**

The study paved the way for the prospective pharmaceutical use of microalgae as an antibiotic replacement therapy or for the extraction of active antimicrobial components.

## 1. Introduction

Endometritis or postpartum infection causes high economic losses in farm animals, as it prolongs the calving interval, decreases the calf crop, and milk yield [1]. Endometritis, either clinical or subclinical, stands behind repeated implantation failure and recurrent miscarriage in up to 40% of infertile patients [2]. The incidence of endometritis or postpartum infection in women is 8-fold higher after caesarean section (CS), which presents 52–65% of cases in Egyptian women [3]. Endometritis is most probably caused by bacterial pathogens such as *Mycoplasma, Streptococcus, Enterococcus, Staphylococcus*, and *Enterobacteriaceae*. These bacterial infections induce oxidative stress, which affects multiple physiological processes in the female reproductive tract, including oocyte development, fertilization, conception, embryo development, and parturition [4]. The expression levels of *IL6, IL10*, and *IL1RA* genes were elevated during the early stages of endometritis in susceptible animals compared with those in unsusceptible animals [5].

*Spirulina platensis* (SP) and *Chlorella vulgaris* (CV) are well-known microalgae that are rich in minerals, vitamins, and protein without any undesirable effect on human and animal health. They are rich in phenolic compounds and have high antioxidant and antimicrobial activity [6, 7]. *Spirulina* and *Chlorella* stimulate innate immune cells, enhancing monocytes and macrophages to produce *TNFα, IL-1β*, and *INFγ*, inflammatory cytokines [8, 9]. Also, daily Spirulina consumption by patients led to elevated *INFγ* expression for 2–3 months after cessation of administration [10, 11]. At the same time, aqueous extracts of CV inhibit other inflammatory mediators and cytokines [6].

This study was based on previous studies that demonstrate the antioxidant and anti-inflammatory effects of SP and CV microalgae. This investigation demonstrates the roles of SP and CV in controlling uterine infection with pathogenic *E. coli* O157 by altering the immunological state of female albino rats. In addition, the expression levels of selected genes and the pathological findings were evaluated.

## 2. Materials and Methods

### 2.1. Ethical approval

The animal experiment was designed and conducted in accordance with the ethical guidelines of the NRC’s ethical committee. The study was reported in accordance with ARRIVE guidelines (ethical approval NO. 20170). The microalgae *Spirulina platensis* and *Chlorella vulgaris* were provided by the microalgae production unit at the National Research Centre, Cairo, Egypt.

### 2.2. Preparation of flavoured microalgae microcapsules

Microalgae leaves were dried at 45°C and mixed with maltodextrin, soy lecithin, and orange-peel oil to improve the microalgae’s unfavourable taste. The maltodextrin and soy lecithin were used in amounts (2:1 w/w) as wall material for encapsulation of the microalgae. Orange peel oil was used as a natural flavour. After magnetic stirring, the entire mixture was homogenized in a Shaker for 30 min at 250 rpm mechanical agitation [12].

Microencapsulation was performed using a Spray Drying apparatus (A BÜCHI, B-290, Switzerland) with a suction rate of 85.0% and inlet and outlet temperatures of 170.0°C (± 1.0°C) and 95.0°C (± 1.0°C), respectively. The powder obtained from the cyclone and drying chamber contains a 1:10 microalgae-to-encapsulation wall ratio [12].

### 2.3. Zeta sizer and morphology of microcapsules

Using a Zeta Sizer Nano ZS (Malvern Instruments Inc., Southborough, MA), the hydrodynamic diameter of the samples was estimated. The morphology of the microalgae powder was assessed by transmission electron microscopy (Quanta FEG 250, FEI, Czech Republic).

### 2.4. Estimation of scavenging activity

Using 1,1-diphenyl-2-picrylhydrazyl (DPPH•), the microalgae’s free radical scavenging activity was measured relative to ascorbic acid as a standard [13].

### 2.5. Estimation of micronutrients of microalgae

The extracts were analyzed by atomic absorption and HPLC-MS.

### 2.6. Antimicrobial assay of microalgae extract

The antibacterial activity of the microalgae extracts (*Spirulina platensis* and *Chlorella vulgaris*, 5 gm crude extracts in 100 ml of dH_2_O) was investigated by the well diffusion method. Four bacterial species were used: *Pseudomonas aeruginosa* ATCC 27853, *Escherichia coli* O157 ATCC 25922, *Bacillus subtilis* ATCC 11778, *Staphylococcus aureus* ATCC 25923, and two fungal species, *Aspergillus Niger* and *Candida albicans* ATCC 14053. Bacterial concentrations (0.5 McFarland standards) were on the Mueller-Hinton agar (MHA) and broth (Difco Laboratories, Detroit, USA). Using potato dextrose agar (PDA) media for 7 days to grow fungus at 27°C. One hundred microliters of each extract were placed in triplicate petri dishes. For bacterial strains, incubation lasted for 24 h at 37°C, while for fungi, it lasted for 48 h at 25°C. Finally, the inhibition zone diameter (mm) was measured and compared for antibiotics and crude oils.

### 2.7. Biological study

#### 2.7.1. Diet formulation

The basal diet was formulated according to the specifications in Table 1. The basal diet was mixed with either SP or CV, and the resulting mixture was adjusted to 1% of the diet.

**Table 1.** Diet formula.

Ingredients	Amount (gm/Kg diet)
Casein	120
Sucrose	110
Oil	50
Salt mixture	35
Vitamin mixture	10
L-cysteine	1.8
Choline chloride	2.5
Starch	660.7
Microalgae (*Spirulina* or *Chlorella*)	10

#### 2.7.2. Animal design

Animal care and treatment throughout the experiment were carried out in accordance with the approval and guidelines of the Institutional Ethical Committee for Medical Research of the National Research Centre, Egypt (Code No: 12-115). This study was approved by the Institutional Ethical Committee for Medical Research of the National Research Centre, Egypt. The study was reported in accordance with the Animal Research: Reporting of *in vivo* Experiments (ARRIVE) guidelines (ethical approval no. 20170). 45 adult female Sprague-Dawley rats were divided into three groups (G1, G2, G3), with 15 rats per group. G1 ate a control basal diet (CD), G2 received CD mixed with SP, and G3 was supplemented by CD mixed with CV.

After 20 days of supplementation, animals were infected with a pathogenic strain of *E. coli* O157 (intra-vaginal injection to induce endometritis using 10^6^ colony count). Vaginal swabs were collected every other day for 10 days to estimate colony counts and monitor recovery rates in different groups of animals. Animals were fasted overnight, euthanized under anaesthesia with ketamine hydrochloride (45 mg/kg, IM) [14]. Blood samples were collected for biochemical analysis using commercial reagent kits from Roche Diagnostics (Laval, QC, Canada). Uteruses were collected, weighed, fixed in 10% formalin-saline, and sent for histology and immunohistochemistry. Uterine tissues were stored at -80°C for the quantification of targeted genes, including *IL-1β, IL-6, TNF-α*, and *INFγ*.

#### 2.7.3. Histopathological examination

Following the collection of samples from the uterus, ovaries, and spleen, fixation and staining of sections of paraffin blocks with Haematoxylin and Eosin for histopathological examination were performed [15].

#### 2.7.4. Histomorphometry of ovaries

Microscopically counting of rat ovarian follicles from three sections of each ovary was estimated using version 6.2.4.5 software of TS View [16].

#### 2.7.5. Histopathological lesion classification

Changes in uterine and spleen tissues were classified according to the degree of severity into four categories as follows: No changes (0), mild (1), moderate (2), and severe (3) changes [17].

#### 2.7.6. Immunohistochemistry

Firstly, ovarian, uterine, and spleen tissue sections were deparaffinised as described by [18]. Secondly, these sections were pre-treated with citrate buffer for 20 min. Tertiary, incubation of these sections for 2 h with anti-*TNF-α* (1/100, Celltech Ltd, UK), then with goat anti-rabbit IgG H&L (HRP) (ab205718; Abcam, Cambridge, UK). Finally, the slides were counterstained with haematoxylin and mounted with DPX. The negative control slides were prepared by replacing the primary antibodies with PBS.

#### 2.7.7. Evaluation of TNF-α immunostaining

*TNF-α* immunoreactivity was quantified in 10 high-power (400×) microscopic fields per section, as described in [19]. The Colo deconvolution image J 1.52 p software (Wayne Rasband, National Institutes of Health (U.S.A.) of stained cells percentage is used as an indicator of a positive result.

#### 2.7.8. Quantitative real-time PCR (qRT-PCR) analysis

Total RNA was extracted from uterine tissues using RNeasy mini kit (Qiagen, Hilden, Germany) (Cat. NO.: 74104). The total RNA was isolated according to the manufacturer’s protocol. Total isolated RNA was subsequently stored at –80°C. The purity and concentration of total RNA were estimated by the NanoDrop One Microvolume UV spectrophotometer 2000/c (Thermo Fisher Scientific, Wilmington, USA). Afterwards, the Revert Aid First Strand cDNA Synthesis Kit (Thermo Fisher Scientific, Waltham, MA, USA) (Cat. NO.: K1621) was used to convert the RNA from each treatment to first-strand cDNA in accordance with the manufacturer’s instructions.

Table 2 illustrates specific primers for the following genes: *TNF-α, IL-1β, IL-6*, and *INF-γ*. The real-time PCR reaction was carried out using Maxima SYBR Green qPCR Master Mix (2×) (Thermo Fisher Scientific, USA) (Cat. NO.: K0221) [20]. The reaction was performed under the conditions of 95°C for 10 min, 95°C for 15 sec, 55°C for 30 sec, and 72°C for 30 sec with a total of 40 cycles of amplification. At the end of the analysis, the melting curve was analyzed to assess amplification specificity. Quantitation of gene expression levels was performed using the DNA Technology Detecting Thermocycler DT Lite 4S1 (Russia).

**Table 2.** Primers used in qRT-PCR analysis.

Gene	Forward primer (5′-3′)	Reverse primer (5′-3′)	References
*β-actin*	CAC GTG GGC CGC TCT AGG CAC CAA	CTC TTT GAT GTC ACG CAC GAT TTC	[21]
*TNF-α*	TAT GGC CCA GAC CCT CAC A	GGA GTA GAC AAG GTA CAA CCC ATC	[22]
*IL-1β*	TCC AGG ATG AGG ACA TGA GCA C	GAA CGT CAC ACA CCA GCA GGT TA	[23]
*IL-6*	CCA CTT CAC AAG TCG GAG GCT TA	CCA GTT TGG TAG CAT CCA TCA TTT C	[22]
*IFN-γ*	TGA GCC AGA TTG TTT CGA TG	TCC TTT TGA AAC TCG GAG GA	[24]

### 2.8. Statistical analysis

The findings were displayed as mean ± SEM. SigmaPlot version 11 is used to analyze the data. The Tukey-Kramer multiple-comparison test and one-way analysis of variance (ANOVA) were used to identify statistically significant differences among groups in the study. To analyze the data, GraphPad Prism version 8 was utilized. A significant difference was considered as *p* < 0.05 for all tests.

## 3. Results and Discussion

### 3.1. Characterization of microcapsules

#### 3.1.1. Particle zeta potential and size of microcapsules

**Figure 1.**
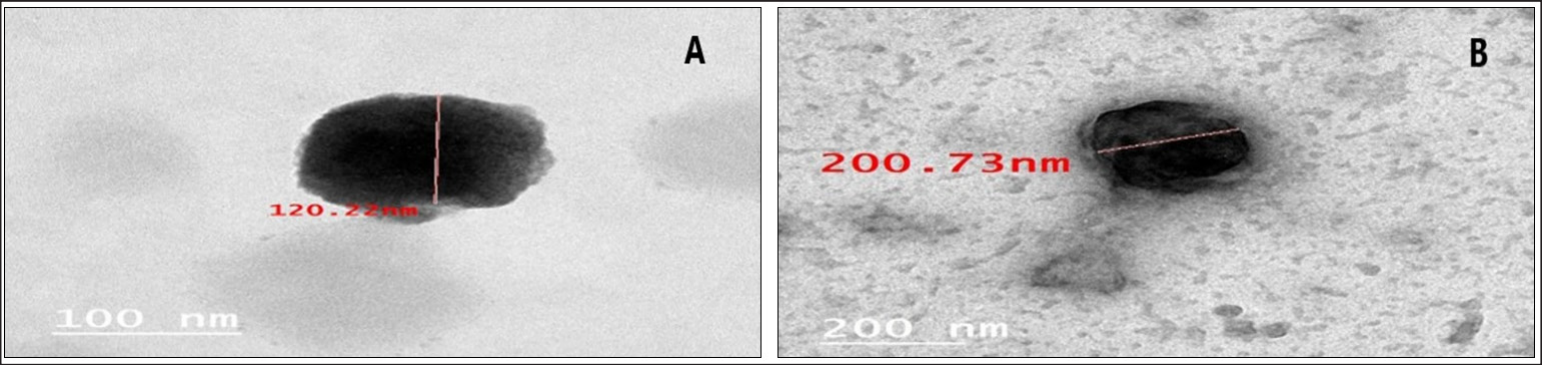
TEM of encapsulated flavored *Chlorella* (A) and *Spirulina* (B).

The morphology of the microcapsules was estimated using a transmission microscope, as shown in Figure 1. There is variance in the physical stability of the microcapsules; they form a strong colloidal system with a ζ-potential (positive or negative), do not aggregate, and repel each other. The values of the ζ-potential in SP and CV were –30.3 and –34.2, respectively, indicating interactions between charged molecules [25]. When the ζ-potential of the particles is higher than 30 mV, they are considered physically stable [25, 26, 27].

#### 3.1.2. Micronutrients in microalgae

Microalgae include not only prokaryotic cyanobacteria (blue-green algae) but also eukaryotic microorganisms and are now considered a source of food and energy. Analysis of microalgae microcapsules (Table 3) showed that they are a rich source of minerals, trace elements, and vitamins. SP was superior in iron, magnesium, calcium, phosphorus, and vitamin A, while CV was superior in Zinc, copper, vitamin E, and D [28].

**Table 3.** Micronutrients of microalgae.

Parameter	*Spirulina*	*Chlorella*
Zinc(mg/kg)	14.5	84.5
Copper mg/kg	1.5	32
Selenium mg/kg	1.25	1
Fe (mg/kg)	220	165
Mg (mg/kg)	1050	675
Ca (mg/kg)	1875	875
Pi (mg/kg)	3500	2750
Vit E (IU)	58	3470
Vit D3(IU)	1427	3040
Vit A(IU)	4559	1639

#### 3.1.3. Antimicrobial activity of microalgae extracts

**Figure 2.**
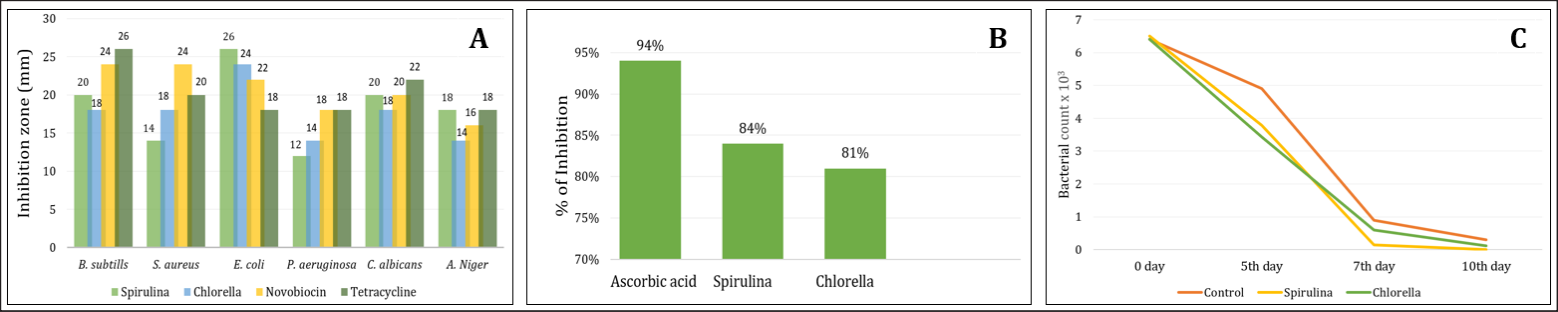
Antimicrobial activity (A), antioxidant effect DPPH (B) of microencapsulated *Spirulina* and *Chlorella*. Rate of uterine clearance(C) of infected animals given SP and CV. Data are represented as mean ± SEM (n=6). A one-way ANOVA was used, followed by the Tukey-Kramer multiple-comparison test.

Results of Figure 2A showed considerable antimicrobial activity against both types of bacteria (Gram-positive and Gram-negative), *Candida albicans*, and *Aspergillus niger*. The mechanism of Microalgae is not through a direct bactericidal effect on the host, but rather by enhancing the animal’s immunity and, consequently, inhibiting the pathogen. Similar findings have been reported in previous studies [7]. The presence of bioactive compounds in the composition of SP and CV, such as diacylglycerols, alkaloids, lipopolysaccharides, cyclic peptides, sulfoquinovosyl, and γ-linolenic acid, contributes to their antimicrobial properties. Also, Chlorellin, isolated from Chlorella species, is a fatty acid that inhibits the growth of both gram-positive and gram-negative bacteria [29, 30]. Novel compounds have also been reported from cyanobacteria and microalgae, and one, EMTAHDCA, exhibits antimicrobial activity against susceptible bacteria. Also, SP and CV have previously shown clear, positive effects against many viruses and fungi [31]. Sulfolipids derived from microalgae form SQDG18 (synthetic sulfoglycolipid), which was used in treatment and vaccination due to its role in upregulating *IL-1α, IL-1β, IL-18*, and *IL-27* genes [29]. Adding *C. vulgaris* to the patient’s diet elevated levels of some inflammatory markers in serum samples, including *IFN-γ, IL-1*, and *IL-1*2, and improved NK activity [32].

#### 3.1.4. Determination of radical DPPH scavenging activity

Because of their high antioxidant content (pigments, peptides, polyphenols, and polysaccharides), microalgae have been used in the cosmetic industry and for pharmaceutical purposes. The present study, shown in Figure 2B, showed that SP and CV extracts exhibit higher antioxidant power (DPPH) than ascorbic acid. Earlier studies reported that SP is rich in Ferulic acid, which is converted to ferulic acid esterase in blood, with antioxidant and antimicrobial activity [32]. Also, the current results declared that CV and SP are rich sources of vitamins, minerals (Table 3), and bioactive substances, which were well known to have numerous physiological activities and antioxidant effects. Many components of algae contribute to their acquired antioxidant capacity, like proteins, polysaccharides, pigments, and polyphenols [7].

### 3.2. Biological study

#### 3.2.1. Bacterial colony count during infection

**Figure 4.**
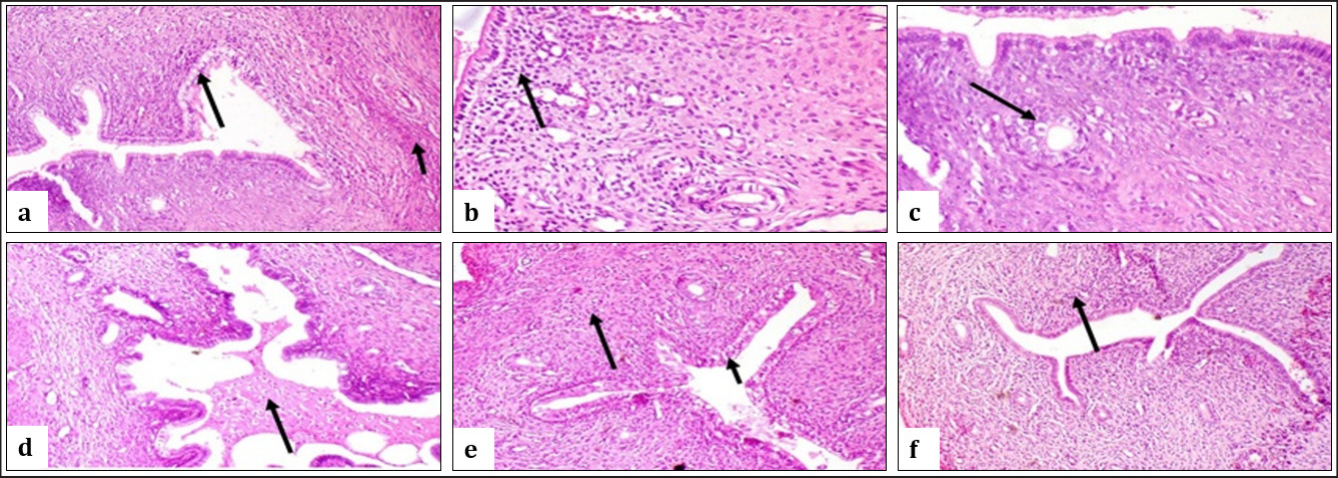
Photomicrograph, rat uterus (a) Control group showing vacuolar degeneration of endometrial lining epithelium (long arrow) and appearance of inflammatory cells in myometrium (short arrow) (b) Control group illustrating numerous inflammatory cells in endometrium (arrow). (c) Control group showing vacuolar degeneration of the lining epithelium of endometrial glands (arrow). (d) Control group showing presence of exudate mixed with desquamated cells inside the uterine lumen (arrow). (e) The group received *Spirulina* showing mild vacuolar degeneration of endometrial epithelium (short arrow) and mild endometrial inflammatory cells infiltration (long arrow). (f) Group treated with *Chlorella* showing moderate inflammatory cell infiltration in the endometrium (arrow). (H&E x200).

The results in Figure 2C showed that regular uterine swabs from infected animals administered CV and SP mixed with the diet had a marked decrease in colony count compared with the control-infected group. This was attributed to the higher antimicrobial activity of SP and CV against *E. coli* than tetracycline and novobiocin, as shown in Figure 2A. After 10 days of infection, the SP group had almost completely recovered, as confirmed by histopathological examination (Figure 4). An earlier study reported that screening for antimicrobial activity of *S. platensis* showed the highest activity against *E. coli* [29] compared with tetracycline antibiotics. The presence of carotenoids and unsaturated fatty acids in microalgae constituents contributes to increasing their bacteriostatic or bactericidal activity [7]. Also, when *C. vulgaris* was mixed into a fish diet at 1.0 gm/kg, it showed the Highest antimicrobial lysozyme activity, better growth performance, and maximum fish survivability [33].

### 3.3. Biochemical analysis of blood plasma

#### 3.3.1. Inflammatory cytokines

**Figure 3.**
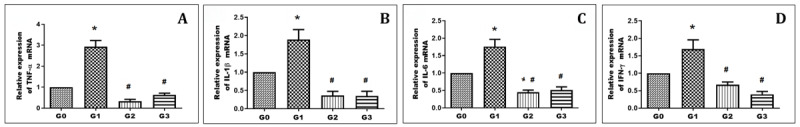
(A-D): Effect of *Spirulina* and *Chlorella* on mRNA expression of inflammatory cytokine genes: (A) *TNF-α*, (B) *IL-1β*, (C) *IL-6*, and (D) *IFN-γ*. Data are represented as mean ± SEM (*n* = 6). ^*^, Versus control non-infected rats at *p* < 0.05. ^#^, Versus control infected rats at *p* < 0.05. A one-way ANOVA was used, followed by the Tukey-Kramer multiple-comparison test.

Concerning the plasma levels of cytokines, Table 4 declared significant elevation in plasma cytokines (*TNF-α, INFγ, IL1β*), especially those administered SP and CV, while *IL-6* level dropped in treated animals. These results were confirmed by quantitative gene expression analysis of uterine samples at the end of the experiment (Figures 3A–D).

**Table 4.** Level of plasma cytokines of infected animals.

Cytokines	Treatment	Before infection	During infection	After recovery
*IL-1β* (Pg/ml)	control	506.82+10.57^a^	540. 28 ± 13.6^b^	530.49+3.78^c^
	SP	520.49+3.78^a^	570.49+3.78^b^	518.52 ± 11.77^c^
	CV	510.49+3.78^a^	550.49+3.78^b^	520.44+38.69^c^
*INFγ* (Pg/ml)	control	94.0 ± 26^a^	140.0 ± 17^a^	132 ± 4.5^a^
	SP	115.0 ± 49^b^	160.0 ± 34^a^	124 ± 13^b^
	CV	118.0 ± 3^a^	184.0 ± 29^b^	122.8 ± 2.8^a^
*IL-6* (Pg/ml)	Control	21.6 ± 1.8^a^	29.4 ± 0.3^a^	26.4 ± 2.0^a^
	SP	16.6 ± 0.9^b^	18.6 ± 2.2^ab^	10.4 ± 1.8^c^
	CV	17.8 ± 0.9^ab^	9.4 ± 1.8^c^	8.7 ± 0.9^b^
*TNFα* (Pg/ml)	Control	29.0 ± 4.7^a^	87.8 ± 8.1^b^	69.6 ± 14^ab^
	SP	36.0 ± 1.26^a^	72.0 ± 11.7^b^	43.6 ± 3.5^b^
	CV	48.2 ± 3.2^b^	106.2 ± 9.1^a^	50.8 ± 4.1^a^

Different superscripts within rows mean a significant difference (*p* ≤ 0.05). Data are represented as mean ± SEM (*n* = 6).

Exposure of animal body systems to external and internal infections enhances their immune response, not only antibody production but also macrophage activation, depending on the Th1/Th2 balance. In this study, high production of *TNF-α, INFγ*, and *IL1β* in rats fed *Spirulina* and *Chlorella*, even before infection, was observed, indicating a mechanism of immune response through Th1-type cell-mediated immunity [34]. This outcome is parallel with other studies demonstrating that mice fed with *Spirulina* had elevated Th1 bias [32]. Other researchers reported that *Spirulina* enhanced *IFN-γ* production by NK cells in response to *IL-12* and *IL-18* when added to human mononuclear cell culture media, and that *Spirulina* extracts were consumed in the diet by human volunteers for 2 months [10]. Also, microalgae exerted anti-inflammatory effects by downregulating the proinflammatory cytokine *IL-6* [9, 35].

#### 3.3.2. Plasma chemistry

**Figure 5.**
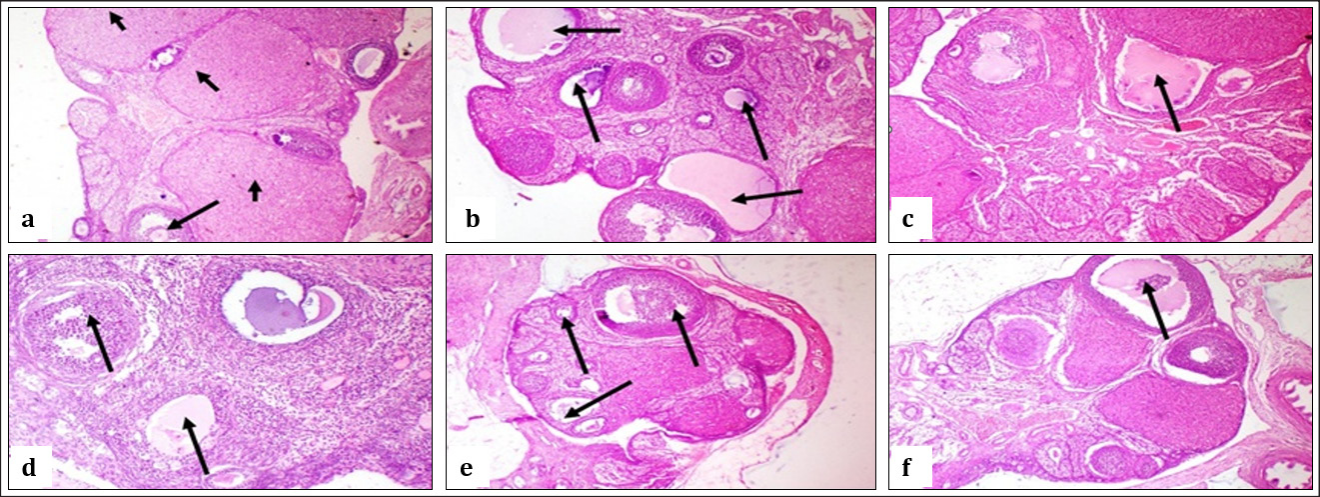
Rat ovaries by photomicrography (a) Control group showing few mature follicles (long arrow) with increased corpora lutea (short arrows) (b) Control group showing presence of multiple ovarian cysts (arrows) (c) Group treated with *Spirulina* showing follicular cyst (arrow) (d) Group treated with *Spirulina* showing presence of ovarian follicles (arrows)(e)Group treated with *Chlorella* showing ovarian follicles (arrows) (f) Group treated with *Chlorella* showing follicular cyst (arrow). (H&E x40)

The current study (Tables 5, 6) revealed that treatment of rats with either *Spirulina* or *Chlorella* did not induce any adverse effects on organ function, glucose levels, or oxidative markers. These results were in coincidence with earlier studies [9, 35]. Regarding sex hormone levels, FSH and estrogen showed significant elevations in the groups administered the microalgae; this was confirmed by examination of ovarian tissue (Figures 5D, C), which showed increased ovarian follicles. Previous studies reported that SP supplementation showed elevated FSH and estrogen levels and enhanced ovarian folliculogenesis [9, 36].

**Table 5.** Effect of Microalgae on plasma profile of female albino rats.

Groups	Control G1	Spirulina G2	Chlorella G3
Glucose (mg/dl)	84.9 ± 10.6^a^	78.17 ± 10.8^a^	137 ± 19.7^b^
Urea (mg/dl)	51.3 ± 2.1^a^	55.6 ± 3.88^a^	31.59 ± 7.0^b^
Creatinine (mg/dl)	1.79 ± 0.01^a^	1.98 ± 0.19^b^	1.64 ± 0.10^a^
Uric acid (mg/dl)	6.18 ± 0.46^a^	6.07 ± 1.05^a^	6.31 ± 0.19^a^
Albumin (mg/dl)	4.74 ± 0.78^a^	3.86 ± 0.62^a^	5.86 ± 1.43^a^
GGT (IU/l)	88.21 ± 4.2^a^	70.43 ± 2.1^b^	30.31 ± 1.8^c^
ALT (IU/l)	22.33 ± 4.78^a^	39.9 ± 9.6^a^	35.86 ± 2.75^a^
Catalase (mmol/mg)	59.62 ± 3.4^a^	56.21 ± 2.03^a^	57.01 ± 2.8^a^
MDA (nmol/ml)	2.4 ± 0.04^a^	1.5 ± 0.02^b^	1.6 ± 0.05^b^
Total protein (mg/dl)	6.44 ± 0.50^a^	5.76 ± 0.62^a^	5.67 ± 0.77^a^

Different superscripts within rows mean a significant difference (*p* ≤ 0.05). Data are represented as mean ± SEM (*n* = 6).

**Table 6.** Effect of microalgae on hormones of infected animals

Groups	Control G1	Spirulina G2	Chlorella G3
FSH (ng/ml)	9.16 ± 0.02ᵃ	11.92 ± 0.01ᵇ	10.01 ± 0.01ᵇ
LH (ng/ml)	9.02 ± 0.31ᵃ	9.93 ± 0.25ᵃ	9.83 ± 0.33ᵃ
Estrogen (pg/ml)	47 ± 0.083ᵃ	54 ± 0.017ᵇ	50 ± 0.011ᵇ
Ghrelin (IU/l)	0.94 ± 0.021ᵃ	0.98 ± 0.008ᵃ	0.85 ± 0.03ᵃ

Different superscripts within rows mean a significant difference (*p* ≤ 0.05). Data are represented as mean ± SEM (*n* = 6).

#### 3.3.3. Quantitative analysis of inflammatory cytokine mRNA expression

Quantitative mRNA expression detection revealed that control group still have over expression of genes (*TNF-α, IL-1β, IL-6*, and *INF-γ*) that responsible for inflammatory response, while the microalgae supplemented groups showed significant lower expression indicating higher recovery rate (*p* < 0.05) in comparison with the infected group as shown in Figure 3A–D, and this result was proved by the immunohistochemistry results of uterine, ovarian and spleen tissue samples (Figure 7). Earlier studies reported that *Spirulina* contains phycocyanin, which acts as an anti-inflammatory by inhibiting pro-inflammatory cytokines released during the early stage of infection [37]. *Chlorella* contains ergosterol that suppresses the inflammatory response of lipopolysaccharide by lowering pro-inflammatory cytokines [38]. Also, the β-carotene present in microalgae downregulates NF-κB, thereby triggering immune and inflammatory responses. Moreover, β-carotene suppresses cytokine production in macrophages, including *IL-β1, IL-6*, and *IL-12*, which are regulated by *IFN-γ* [39]. According to a recent study, β-carotene can suppress NO, prostaglandin E2, and superoxide dismutase production, down-regulate iNOS/COX/NADPH expression, and suppress *TNF-α* to achieve anti-inflammatory effects [40].

### 3.4. Histopathological examination

#### 3.4.1. Uterus

Examination of uterine tissue showed that the infected control group had vacuolar degeneration of endometrial lining epithelium and infiltration of myometrium with inflammatory cells as shown in Figure 4a, heavy infiltration of endometrium with inflammatory cells as shown in Figure 4b, vacuolar degeneration of lining epithelium of endometrial glands as shown in Figure 4c, also there was exudate mixed with desquamated cells and inflammatory cells inside uterine lumen as shown in Figure 4d. Mice that received *Spirulina* showed mild, clear degeneration of the endometrial epithelium with vacuoles, and mild infiltration of endometrial inflammatory cells, as shown in Figure 4e. Group treated with *Chlorella* showed moderate infiltration of endometrial inflammatory cells, as shown in Figure 4f (H&E x200).

#### 3.4.2. Ovaries

The control group showed few mature follicles and increased corpora lutea (Figure 5a), with multiple ovarian follicular cysts (Figure 5b). The group treated with *Spirulina* showed few follicular cysts in Figure 5c and multiple mature ovarian follicles in Figure 5d. Group treated with *Chlorella* showed mature ovarian follicles (Figure 5e), with few follicular cysts (Figure 5f).

#### 3.4.3. Spleen

**Figure 6.**
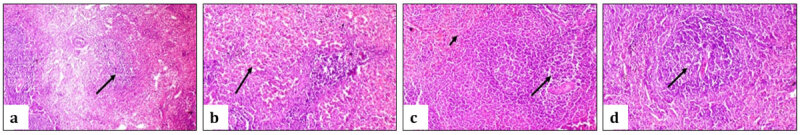
Photomicrograph, rat spleen (a) Control group showing lymphoid depletion in white pulp(arrow) (b) Control group showing clear hemorrhage in splenic tissue (arrow) (c) Group received *Spirulina* showing nearly normal lymphoid follicle (long arrow) with mild hemorrhage (short arrow) (d) Group treated with *Chlorella* showing mild lymphoid depletion of lymphoid follicle (arrow). (H&E x200).

Examination of spleen tissues showed that the Control group showed lymphoid depletion in the white pulp in Figure 6a and splenic haemorrhage in Figure 6b. Group treated with *Spirulina* showed normal lymphoid follicles and mild splenic haemorrhage in Figure 6c. Group treated with *Chlorella* showed mild lymphoid depletion of lymphoid follicles in Figure 6d.

### 3.5. Histomorphometric findings of ovaries

By counting the number of follicles between different treated groups, there was a significant difference in the number of mature follicles and follicular cysts, as shown in Table 7. Earlier studies showed that 70% of animals suffering from endometritis showed ovarian acyclicity and dysfunction [3].

**Table 7.** Effect of microalgae on the number of ovarian follicles and follicular cysts.

Groups	No. of ovarian follicle	No. of follicular cysts
Control	3.25 ± 1.04^a^	5.75 ± 0.99^a^
*Spirulina*	7.82 ± 2.26^b^	1.72 ± 1.51^b^
*Chlorella*	5.28 ± 3.12^c^	2.13 ± 0.57^c^

The results are expressed as the means ± SEM, where *n* = 10. Superscript refers to significance. *p* ≤ 0.05 was considered significant.

### 3.6. Histopathological findings

Histopathological changes and the severity of lesions in the uterine and spleen tissues were recorded in Table 8. These findings confirm that SP has achieved a higher recovery rate, as shown in Figure 4.

**Table 8.** Histopathological lesions scoring in the uterus and spleen.

Lesion	Control	*Spirulina*	*Chlorella*
Uterus			
Inflammatory cell in the endometrium	3	1	2
Vacuolar degeneration of the endometrial lining epithelium	3	1	2
Spleen			
Lymphoid depletion	3	0	1
Splenic hemorrhage	3	1	1

Score 0 = absence of the lesion in all rats of the group (*n* = 6), score 1 = (< 30%), score 2 = (≥ 30% – 50%), score 3 = (> 50%). ICE, Inflammatory cell in the endometrium; VDELE, Vacuolar degeneration of endometrial lining epithelium; LD, Lymphoid depletion; SH, Splenic hemorrhage.

### 3.7. Immunohistochemical findings for TNF-α

**Figure 7.**
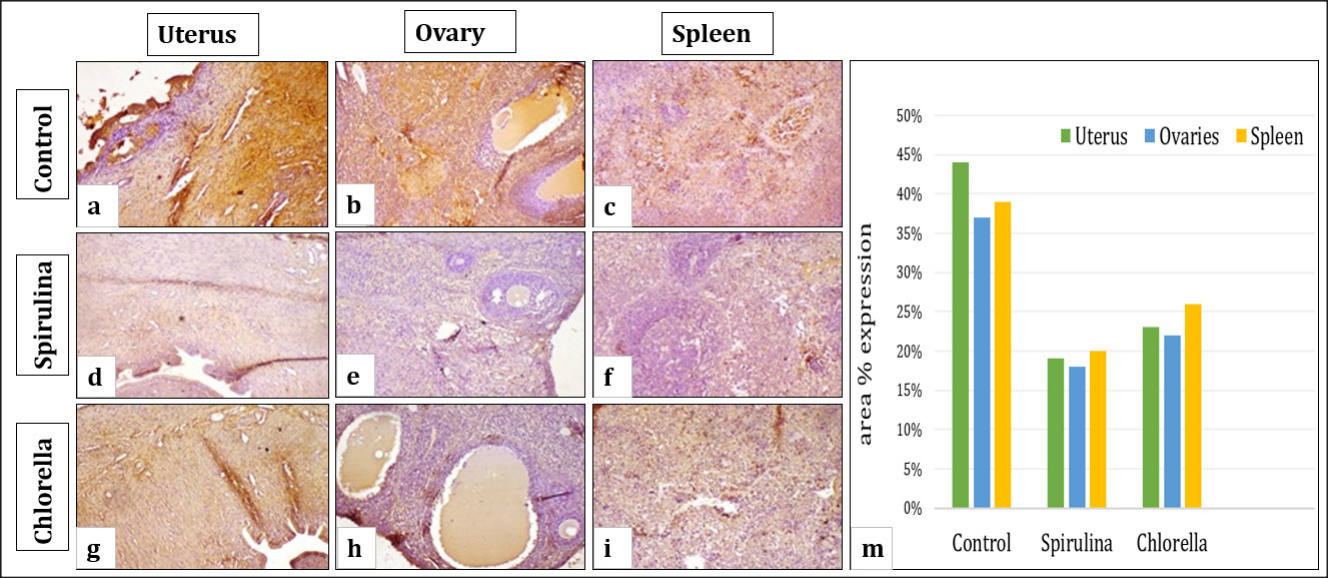
Immunostaining of *TNF-α* in the studied organs (uterus, ovaries, and spleen). (a-c) The control group demonstrates strong immunoreactivity. (d-f) The group received *Spirulina*, showing low immune expression. (g-i) Group treated with *Chlorella* showing moderate expression of *TNF-α*. (*TNF-α* X200) (m)Area % expression of *TNF-α* in uterus, ovaries, and spleen (data were expressed as mean ± SE (*n* = 6), different letters indicating significant differences at *p* < 0.05). A one-way ANOVA was used, followed by the Tukey-Kramer multiple-comparison test.

*TNF-α* Immunostaining expression in the uterus, ovary, and spleen of different treated groups is shown in Figure 7. Immuno-staining of the control group revealed strong expression of *TNF-α*, as shown in Figures 7a, b, c. Earlier studies reported that chronic endometritis causes oxidative stress and inflammation in the ovaries and other tissues [3]. The group treated with *Spirulina* showed a weak positive reaction in Figures 7d, 7e, 7f. The group treated with *Chlorella* showed a moderate positive reaction in Figures 7g, h, i. These findings confirmed the current results found in serum cytokines, as shown in Table 4, and mRNA expression in Figure 3A–D, and the antimicrobial effect of Chlorella against *E. coli* is less than that of *Spirulina*, as declared in Figure 2.

## 4. Conclusions

SP and CV microcapsules showed high anti-inflammatory, antioxidant, and antimicrobial activity. Analysis of microalgae showed their high nutritive value and the presence of valuable pharmaceutical compounds, which achieved a high recovery rate in endometritis in rats through bioactive compounds in their structure that promote endometrial cell growth and repair, support ovary and spleen recovery, and strengthen the immune system as immunostimulants. In summary, this study highlighted the high potential of SP and CV microalgae for use as an antibiotic replacement therapy, with the possibility of large-scale production and extraction of valuable pharmaceutical components. Also, the formulated microcapsules and the added flavor greatly improved the unpleasant fishy taste of SP and CV for consumers.

## Data Availability

The data presented in this study are available from the corresponding author upon reasonable request.

## List of abbreviations

SP, *Spirulina platensis*; CV, *Chlorella vulgaris*; G1, group1; G2, group2; G3, group3; CD, control basal diet; *TNF-ᾳ*, tumor necrosis factor alpha; *IL1β*, interleukin1beta; *IL6*, interleukin 6; DPPH, 1.1-diphenyl-2-picryl-hydrazyl; *INF-γ*, interferon gamma; CS, caesarean section; MHA, Mueller Hilton agar; PDA, potato dextrose agar.
